# Increased gut permeability in newborns with food allergy

**DOI:** 10.1186/2045-7022-3-S3-P94

**Published:** 2013-07-25

**Authors:** S Makarova, T Borovik, V Skvortsova, G Yatsyk, I Gmoshinskij, V Mazo

**Affiliations:** 1 Scientific Center of Children's Health, RAMS, Moscow, Russian Federation; 2 Institute of Nutrition, RAMS, Moscow, Russian Federation

## Background

Gut barrier condition is very important factor for development of food tolerance. In the first few years of life, humans gradually develop an intricate balance between tolerance and immune reactivity in the gut mucosa.

Increased absorption of food antigens in early childhood may play role for sensitization and development of food allergy in the future. The purpose of the study is investigation of gut permeability in newborn infants with first symptoms of allergy.

## Methods

51 breastfed newborns were under examination. Human milk α-lactalbumin was measured in blood serum by ELISA after breast milk feeding. Index of absorption was calculated according to the amount of consumed breast milk. Gut permeability was assessed twice, at 5-7 and 25-30 days of life.

## Results

Examination atthe age of 5-7days of life revealed that newborns had high level of absorption of human milk α-lactalbumin. At 25-30 days of life non-allergic infants had shown a 4- fold reduction of gut permeability during the first month of life regardless of gestation age and the type of feeding. But, it was found that absorption of human milk α-lactalbumin did not decrease in 60% of infants with allergic symptoms appeared during the first month after birth (р < 0,01) and in 35,7% of malnourish infants with diarrhea syndrome (р < 0,01).

## Conclusion

Patients with early allergic symptoms have increased gut permeability for proteins incomparison to non-allergic infants. So, management of these patients has to include measures to reduce theoral uptake of allergens, such as maternal diet and using of hydrolysed formulas.

## Disclosure of interest

None declared.

